# Empathy in Musicians: Self‐Report Versus Performance on an Empathic Accuracy Task

**DOI:** 10.1002/ijop.70072

**Published:** 2025-07-11

**Authors:** M. aan het Rot, I. M. Venema, M. Franzen, D. Başkent

**Affiliations:** ^1^ Department of Psychology and Research School of Behavioral and Cognitive Neurosciences University of Groningen Groningen the Netherlands; ^2^ Department of Otorhinolaryngology Leiden University Medical Center Leiden the Netherlands; ^3^ Department of Psychology, Education and Child Studies Erasmus University Rotterdam Rotterdam the Netherlands; ^4^ Department of Otorhinolaryngology/Head and Neck Surgery, University Medical Centre Groningen & Research School of Behavioral and Cognitive Neurosciences University of Groningen Groningen the Netherlands

**Keywords:** affective empathy, cognitive empathy, empathic accuracy, musical training, musicality, musician effect

## Abstract

Some studies indicate enhanced vocal emotion recognition and emotional prosody perception in musicians. Music perception has been linked to emotion processing. Collective music making has been found to rely on responding to and sharing the emotions of others. Together, these notions suggest musicians may have more empathy, which constitutes the ability to experience and understand others' emotions. In the present study, we asked 25 professional musicians and 23 non‐musicians to complete the Empathy Quotient (EQ), a self‐report questionnaire of affective and cognitive empathy, and a performance measure of empathic accuracy (EA) that involved watching and listening to video clips of targets narrating emotional autobiographical events. EA was derived per participant per clip by correlating their ratings of how targets felt while talking with previously collected target ratings. While musicians scored higher on both EQ subscales, they did not differ significantly from non‐musicians in EA, obtained using rich stimuli involving both auditory and visual information. Hence, while musicians rated themselves to be more empathetic, we found no objective evidence of a musician benefit in empathy. It remains possible that this may show in less information‐rich or more music‐based situations. Alternatively, factors other than musical training alone may play a role.

## 1 Introduction

Empathy is a multifaceted construct, involving affective and cognitive components (Yu and Chou 2018). Being able to (affectively) experience the feelings of others and to (cognitively) understand them is considered important during social interactions. Feelings can be communicated via auditory and visual cues, such as vocalisations, speech, facial and postural expressions (de Gelder and Vroomen 2000) and also via music (Vuoskoski et al. 2022). Compared to non‐musicians, professional musicians may show different patterns in, and even score better on, vocal emotion recognition (VER; Martins et al. 2021; Nussbaum et al. 2024). While not all studies show this, it could mean that musical training may bring about a VER benefit by improving the hearing and processing of auditory emotion cues. Alternatively, or additionally, musicians may have better VER thanks to an innate disposition for better hearing and listening, which might have contributed to their decision to start musical training (Martins et al. 2021; Schellenberg and Lima 2024). In any case, musical training has been linked to VER and related processes such as emotional prosody perception (Jansen et al. 2023). In light of these links, we examined whether musicians and non‐musicians might also differ on empathy, using both a self‐report questionnaire and a performance‐based measure.

### 1.1 VER and Emotional Prosody Perception in Musicians

When Martins et al. (2021) reviewed 20 correlational studies, the outcomes indicated enhanced VER among musicians. This may be explained by a trained ability to perceive pitch, which carries emotional information in voices (Globerson et al. 2013). Indeed, musical training has been associated with better VER abilities (Martins et al. 2021) as well as with better emotional prosody perception (for a meta‐analysis of relevant studies, see Jansen et al. 2023). This suggests a ‘musician effect’, meaning that musical training might improve VER and emotional prosody perception. Relatedly, musical training has been associated with altered activity in brain areas specialised in the processing of emotions (Vuust et al. 2022).

The potential influence of musical training on VER has been examined in experimental studies. In one study, 43 children were randomly assigned to keyboard, singing or drama lessons, or to no lessons (Thompson et al. 2004). After 1 year, children trained in keyboard (and drama) were better than untrained children at identifying anger and fear in semantically neutral speech, suggesting improved emotional prosody perception. Unfortunately, whether children actually improved remained unclear, as they had not been tested before the lessons. However, another study randomly divided 24 college students, who never learnt to play a musical instrument, into a group receiving four music lessons and a group receiving four art lessons (Mualem and Lavidor 2015). VER, assessed before and after the lessons, only improved in the music group, suggesting that the music lessons improved VER.

Nonetheless, it remains unclear if such an acute effect generalises to a long‐term influence of the extensive training completed by professional musicians. Moreover, even if experimental studies suggest an influence of musical training on VER and emotional prosody perception, to what degree the musical training actually contributes to a ‘musician effect’ remains unclear. Enhanced VER and emotional prosody perception in musicians may also be explained by certain personal characteristics that contributed to them becoming musicians (Schellenberg and Lima 2024). In support of this, while Mualem and Lavidor (2015) found no significant differences between the pre‐intervention VER scores of their non‐musician participants and those of trained musicians, the two groups differed in music perception. Similarly, Correia et al. (2022) studied adults with varying levels of musical training and musical abilities and reported that both VER and emotional prosody perception were weakly positively associated with musical training and moderately positively with (objectively measured) musical abilities, even when controlling for general cognitive abilities. On the basis of these and other studies, Jansen et al. (2023) also concluded that emotional prosody perception was more strongly correlated with music perception abilities than with musical training.

It has been argued that musicians' enhanced ability to perceive emotions in speech may stem from an innate disposition to listen to melody in music (e.g., Nussbaum et al. 2024). The reverse may also be true: people inclined to hearing emotions in others' voices may be more likely to develop the ability to perceive melody, and become musicians (e.g., Schellenberg and Lima 2024). Besides, enhanced VER and emotional prosody perception among musicians could be due to factors other than musical ability. For example, in one study conducted in 100 university students, while VER did not significantly correlate with musical training or musical abilities, there was a positive correlation with emotional intelligence (Trimmer and Cuddy 2008). Musical training may help improve emotional intelligence, and higher emotional intelligence may benefit musical performance (Campayo‐Muñoz et al. 2020). Between‐group differences in emotion processing by the brain (Vuust et al. 2022) may help explain why musicians may have higher emotional intelligence than non‐musicians.

One other potential factor is general intelligence. Schellenberg and Mankarious (2012) found that while musically trained children scored higher on an emotion comprehension test than untrained children, this higher score was strongly related to a higher score on an IQ test. Butkovic and Rancic Dopudj (2017) also found differences between adult musicians and non‐musicians in intelligence, using a self‐report measure of personality. Indeed, personality could be yet another factor explaining enhanced VER and emotional prosody perception among musicians. They have been shown to score higher on extraversion and agreeableness (Butkovic and Rancic Dopudj 2017) and on openness to experience (Gjermunds et al. 2020). However, Lima and Castro (2011) found no significant differences between trained musicians and untrained controls in intelligence, personality, or cognitive control.

Overall, it seems that the question of whether findings of enhanced VER and emotional prosody perception in musicians stem from musical training, innate musical abilities, or even non‐musical abilities has not yet been answered conclusively. A combination of factors may provide the best explanation, with each factor contributing to different degrees in different musicians. This would also help explain why the evidence for enhanced VER and emotional prosody perception in musicians is mixed, as acknowledged by both Martins et al. (2021) and Jansen et al. (2023).

### 1.2 Empathy in Musicians

Another factor that may be associated with enhanced VER and emotional prosody perception among musicians is empathy, which is related to emotional intelligence but primarily concerned with the feelings of others. Empathy is considered important for playing music together and for being able to communicate emotions to audiences (King and Waddington 2017). In a sample of 58 adult non‐musicians, Novembre et al. (2019) found that dyads with higher self‐reported cognitive empathy, assessed using the Perspective Taking subscale of the Interpersonal Reactivity Index (IRI), were more interpersonally synchronous on a collective music‐making task. This finding corresponds with the notion that emotional intelligence may benefit musical performance (Campayo‐Muñoz et al. 2020) and additionally suggests that musicians with more empathy may have a better ability to apply this emotional skill to musical performance. Moreover, collective music making may promote empathy (Cho and Yeoun Han 2022). Indeed, Rabinowitch et al. (2013) found that children in a musical group interaction programme—which primarily aimed to improve empathy, rather than provide musical training—showed enhanced affective empathy over time, as well as in comparison to children not in the programme.

### 1.3 From VER and Emotional Prosody Perception to Empathy

VER and emotional prosody perception both involve the processing of auditory stimuli. However, when people interact, including when communicating emotions, this usually involves an exchange of multiple types of information via both visual and auditory cues (de Gelder and Vroomen 2000). Auditory information may be provided in the form of semantic content (i.e., what was said) and in the tone of voice (i.e., how it was said). Visual information is primarily provided in the form of facial and postural expressions.

Both auditory and visual cues can contribute to empathic responding during social interactions. Concerning visual information, the emotions expressed by a sender's face or posture are, in a receiver, thought to contribute to the emergence of shared affect or feelings of concern, that is, to affective empathy. According to the dual route model of empathy, this happens via a fast, automatic route, while cognitive empathy involves a slower, more effortful route (Yu and Chou 2018). Concerning auditory information, semantic content provided by a sender about their emotional state might primarily activate cognitive empathy in a receiver, because the receiver can use knowledge gained from what the sender said to make inferences about how the sender was feeling. In comparison, emotional prosody in the voice of the sender is more likely to elicit affective empathy (Mauchand and Pell 2022).

The dual route model holds that affective empathy and cognitive empathy can influence each other, thereby impacting someone's overall empathic response (Yu and Chou 2018). Cognitive empathy may only emerge once a receiver has become conscious of a sender's feelings. Thus, affective empathy can influence cognitive empathy. At the same time, the reverse is also thought to occur.

Auditory information in particular is thought to facilitate empathy (Zaki et al. 2009), by communicating emotions either semantically or via tone of voice. Relatedly, music listeners with a stronger emotional response to happy and sad music have been shown to rate themselves higher on affective empathy (Vuoskoski et al. 2022). If listening to music can have an emotional effect, by activating emotion processing areas in the brain (Vuust et al. 2022), and if musicians are good listeners (either innately or by training) as suggested by their enhanced VER and emotional prosody perception, then musicians may generally be more empathetic than non‐musicians. This would fit with the role musician empathy is thought to have during collective music‐making and musical performance (King and Waddington 2017).

### 1.4 The Present Study

Musicians and non‐musicians have been found to differ in their ability to listen to acoustic emotion cues, which includes VER and emotional prosody perception (Jansen et al. 2023; Martins et al. 2021). In the present study, given what is known about the role of auditory information in communicating emotions, we used these findings to hypothesise that musicians and non‐musicians might also differ in their ability to respond affectively or cognitively to such cues, that is, in empathy.

Previous studies on empathy in musicians are rare (Novembre et al. 2019; Rabinowitch et al. 2013) and have mostly used self‐report questionnaires, which are subjective in nature. Moreover, while questionnaires may include items designed to assess both affective and cognitive empathy, they assess empathy as a broad trait rather than at a state level, in the context of specific social situations. In comparison, the empathic accuracy task (EAT; aan het Rot and Hogenelst 2014; Zaki et al. 2008) is a more objective performance measure, designed to examine empathy during situations characterised by a sender expressing emotions while narrating an autobiographical story to a receiver (i.e., a study participant). Importantly, the EAT considers the ability of receivers to recognise positive and negative emotions as well as the emotional expressivity of the senders of these emotions (i.e., the presumed targets of receivers' empathy). Moreover, the EAT presents participants with both visual and auditory information, which makes it the most ‘real‐world’ way of assessing empathy in a controlled lab setting.

Both English and Dutch versions of the EAT have previously been shown to be sensitive to trait‐level differences between study participants (aan het Rot and Hogenelst 2014; Lee et al. 2011; Zaki et al. 2008). Moreover, empathic accuracy (EA) scores have been shown to vary by target emotional expressivity (aan het Rot and Hogenelst 2014; Zaki et al. 2008). While expressivity was assessed by asking targets how easy others can ‘read’ them from visual cues, more expressive targets may also use more auditory cues. These cues, in particular, are thought to facilitate EA (Zaki et al. 2009). Also, these cues may benefit individuals with potentially enhanced VER and emotional prosody perception, such as musicians. Therefore, in the present study, we considered the EAT to be a particularly suitable measure for assessing empathy.

We hypothesised that compared to non‐musicians, musicians would (1) score higher on the Empathy Quotient (EQ), a self‐report questionnaire and (2) perform better on the EAT. These hypotheses were based on previous findings of enhanced VER and emotional prosody perception among musicians (Jansen et al. 2023; Martins et al. 2021). A higher score on the EQ Affective Empathy subscale would also be expected in light of the aforementioned results by Rabinowitch et al. (2013), who focused on affective empathy measures. Similarly, a higher score on the EQ Cognitive Empathy subscale would also be expected in light of the aforementioned results by Novembre et al. (2019).

Additionally, we explored the degree to which the hypothesised group differences in empathy could be attributed to factors other than musical training or innate musical abilities. The NEO Five‐Factor Inventory (NEO‐FFI) is a self‐report questionnaire to assess the Big Five personality traits of agreeableness, conscientiousness, extraversion, neuroticism and openness to experience. Self‐reported empathy has been positively associated with both openness and conscientiousness across studies, with generally more variance explained by openness (e.g., Melchers et al. 2016). Personality traits have also been shown to differ between musicians and non‐musicians, whereby musicians may score higher on openness (Gjermunds et al. 2020). If empathy is higher among musicians, this may at least partially be thanks to this trait.

## 2 Methods

### 2.1 Participants

Forty‐eight participants (37 female, 11 male) were recruited via the Prins Claus Conservatoire in Groningen, and the second author's personal network. The mean age was 25 years (SD = 9, range 18–54 years). Except for one native speaker of Frisian (who spoke Dutch fluently), all participants were native speakers of Dutch. Selection criteria were (1) normal hearing, (2) no past hearing‐related problems that were untreated and (3) self‐reported absence of psychiatric or neurological disorders. Normal hearing was both checked by a questionnaire (see Section 2.2) and also formally screened via normal hearing thresholds (< 20 dB HL) measured at the audiometric frequencies between 250 Hz and 8 kHz.

### 2.2 Measures

#### 2.2.1 Musical Background Questionnaire

This questionnaire was derived from previous work (Fuller et al. 2014) and used to check both the general inclusion criteria and the specific musician inclusion criteria and to characterise participants' musical background and activities. Participants were first asked whether they (a) played an instrument and if so, which one and for how long, and (b) were having singing lessons or singing in a choir. Then, three questions were asked to check the general inclusion criteria (see Section 2.1). Finally, participants were asked several additional questions pertaining to their musical background. Initially, the specific musician inclusion criteria were (a) having started music lessons before the age of 8 years, (b) having had at least 10 years of music lessons and (c) having been musically active in the 3 years prior to the study. However, for feasibility reasons, two participants who started music lessons at the age of 10 years were eventually also included as musicians.

The non‐musicians were individuals who did not meet the aforementioned musician inclusion criteria. While 16 non‐musicians had also had lessons in playing an instrument or singing in the past, all but 2 had been musically inactive in the past 3 years.

#### 2.2.2 Empathy Questionnaire

We used a Dutch translation of the EQ (Groen et al. 2015). This version includes 28 self‐report items divided across three subscales (Affective Empathy, Cognitive Empathy, Social Skills) and 14 distractors. Respondents are asked to indicate their level of agreement with each item (e.g., ‘I find it easy to put myself in someone else's shoes’ for Affective Empathy and ‘I am good at predicting how someone will feel’ for Cognitive Empathy) using a Likert scale ranging from 0 (*completely agree*) to 3 (*completely disagree*).

The Dutch EQ has previously been shown to have good psychometric properties (Groen et al. 2015). Given our hypotheses and in line with previous studies (aan het Rot et al. 2023), we only analysed participants' scores for Affective Empathy and Cognitive Empathy. These subscale scores were generated by taking the sum across 5 positively worded items and 6 negatively worded, reverse‐scored items for Affective Empathy, and across 11 positively worded items for Cognitive Empathy. In our sample, internal consistency was good for the Cognitive Empathy subscale (*α* = 0.83) but questionable for the Affective Empathy subscale (*α* = 0.65).

#### 2.2.3 Personality Questionnaire

To assess the Big Five, we used a previously validated Dutch translation of the NEO‐FFI (Hoekstra et al. 1996). The NEO‐FFI has 60 items, or 12 each for Neuroticism, Extraversion, Openness to Experience, Conscientiousness, and Agreeableness. Each item can be scored from 1 (*completely disagree*) to 5 (*completely agree*). Subscale scores are generated by taking the sum across all relevant items. In our sample, the internal consistency was good for Neuroticism (*α* = 0.87), Extraversion (*α* = 0.82) and Conscientiousness (*α* = 0.80), but questionable for Agreeableness (*α* = 0.64) and poor for Openness (*α* = 0.55). The Openness item with the largest negative effect on the internal consistency was ‘I often try new and foreign foods’.

#### 2.2.4 EAT

A Dutch‐language version of this computerised task was previously developed by aan het Rot and Hogenelst (2014). They found that EA scores were not significantly altered by less expressive targets, indicating the reliability of the target ratings; that EA scores were positively predicted by scores on the Balanced Emotional Empathy Scale, supporting the validity of the EAT; and that EA scores were sensitive to between‐person differences in autism spectrum traits (aan het Rot and Hogenelst 2014).

The Dutch EAT was programmed in E‐Studio 2.0 (Psychology Software Tools). In the original version, the task includes two sets of 20 video clips each. In each clip, one of nine target individuals (four female, five male; mean age = 33 years, range 23–62 years) recalls positive and negative experiences from their past, such as winning an important sporting match or hearing about a relative's illness. In line with previous work (aan het Rot et al. 2023), participants in the present study rated 16 video clips (8 positive valence, 8 negative valence; still from the same 9 targets).

The video clips were on average about 2 min long. Video clip selection was pseudo‐randomised. Participants never saw the same target individual twice in a row, and they never saw more than two clips of the same valence (positive, negative) in a row. More task details are available from aan het Rot and Hogenelst (2014).

The task was presented using E‐Run 2.0 (Psychology Software Tools) on a Windows 7 laptop in a quiet room (e.g., an empty classroom) to prevent distractions, with the built‐in speaker on maximum volume. This way, each target was always audible and presented at a comfortable loudness level. While watching and listening to the video clips, participants were asked to continuously rate the emotional state of the targets using a 9‐point Likert scale presented on‐screen, with 1 (*extremely negative*) and 9 (*extremely positive*) as anchors. These ratings were made using a dial connected to the laptop, which logged them using a score from 0 to 80 in the E‐Prime data file generated for each participant.

During task development, all targets shown in the video clips provided self‐ratings of their own clips in a similar manner (aan het Rot and Hogenelst 2014). In line with previous studies, these self‐ratings were used as a reference for evaluating participant performance. The continuous ratings of both participants and targets were first transformed to a range of −40 to +40 and then averaged across 5‐s intervals for smoothing purposes. After this, in line with aan het Rot and Hogenelst (2014), the first and last 5‐s intervals were discarded (e.g., participants tended to turn back the rating dial to neutral right before the end of a clip). Finally, for each participant/clip combination, the ratings of the participant and those of the target in the clip were correlated, resulting in a correlation coefficient *r* that represents a participant's EA score for a specific video clip.

The maximum number of participant/clip combinations in the EAT dataset was 768. However, one participant saw only seven clips due to a software error. The mean *r*, or EA score, across the remaining participant/clip combinations was 0.65 (range −0.99 to +1.00).

### 2.3 Procedure

First, we provided potential participants with written information about the study. They also received a verbal explanation and were given the opportunity to ask questions. Individuals who verbally agreed to participate were asked to provide written informed consent.

The total study length was less than 60 min. After the hearing screening, participants first completed on paper the musical background questionnaire, EQ and NEO‐FFI, in this fixed order. This took no more than 10–15 min. All questionnaires were checked for missing responses. Before completing the EAT, participants could practise using the rating dial during an instructional video clip. After the completion of the EAT, which lasted around 30–35 min, participants were asked about their experiences, debriefed and given a 10‐euro gift card.

### 2.4 Data Analysis

We used SAS 9.4 software for Windows (SAS Institute, Cary, NC) for all analyses. The *α* was set at 0.05. To test the significance of group differences on the demographic data and questionnaire scores (musical background questionnaire, EQ, NEO‐FFI), we used either *t* tests with Group (two levels: musicians, non‐musicians) as the between‐subjects factor or, for measures with a nominal scale, *χ* ^2^ tests.

The Fisher *z*‐transformation was used on the raw EA scores (*r* values) to create an approximately normal distribution. To answer our research question on group differences in EA, we employed hierarchical linear models with maximum likelihood estimation. Results of these models are reported using estimated least‐squares means and standard errors (SEs).

Before testing our hypothesis, we considered two preliminary hierarchical linear models. This was done because EA may differ for positive versus negative clips and for clips with female versus male targets (aan het Rot and Hogenelst 2014; aan het Rot et al. 2023). One model included only the main effect for Valence (two levels: positive, negative) and the other model only Target gender (two levels: female, male). In line with previous work, in case of a significant main effect for Valence or Target gender, we considered this variable as a covariate in the analyses described below.

Subsequently, we tested our hypothesis that musicians and non‐musicians would differ in EAT performance. First, we entered only the main effect for Group as a predictor of EA. Second, we added the relevant covariates. Thirdly, we examined these covariates as potential moderators of the hypothesised effect of Group. Note that while *z*‐transformed EA scores were used for hypothesis testing and other analyses, raw EA scores (*r* values) are occasionally mentioned for interpretation purposes.

## 3 Results

### 3.1 Demographic, Musical Background and Personality Data

Each participant was assigned into a group as musician (*n* = 25) or non‐musician (*n* = 23). Most musicians played violin (*n* = 11), cello (*n* = 5) or piano (*n* = 4); two were singers and the three remaining each played another instrument (i.e., clarinet, cornet or hobo). Some non‐musicians also played or had played piano (*n* = 6) or cello (*n* = 1) or sang (*n* = 1) while the others played or had played various other instruments (e.g., flute, guitar, saxophone) or not. Table 1 summarises the relevant demographic and other questionnaire data for both participant groups. The age range was 18–54 years for the musicians and 18–51 years for the non‐musicians. The demographic data indicate that while the two groups did not significantly differ in age distribution and gender, they differed in musical background, confirming that the musician inclusion criteria were met. The EQ and Big Five results showed that compared to non‐musicians, musicians self‐rated significantly higher on cognitive and affective empathy, as well as on openness, agreeableness, and conscientiousness.

**TABLE 1:** Demographic, musical background, empathy and personality data by group.

	Musicians (*n* = 25)	Non‐musicians (*n* = 23)	Test of group difference
% Female	76%	78%	*χ* ^2^ = 0.03
Age (years)	26 (9)	24 (9)	*t* = −0.52
% playing a musical instrument or singing	100%	43%	*χ* ^2^ = 19***
% formal music education	60%	0%	*χ* ^2^ = 20***
% frequent music making in the past 3 years	60%	0%	*χ* ^2^ = 20***
Duration of musical training (years)	16 (3)	3 (2)	*t* = −18.41***
Age at start musical training (years)	6 (2)	13 (10)^a^	*t* = 2.78*
Weekly amount of music making (hours)	21 (13)	1 (2)	*t* = −7.19***
Cognitive empathy	16 (3)	12 (3)	*t* = −4.32***
Affective empathy	16 (3)	14 (3)	*t* = −2.19*
Neuroticism	32 (9)	36 (8)	*t* = 1.54
Extraversion	45 (6)	43 (7)	*t* = −0.91
Openness	44 (4)	40 (5)	*t* = −2.55*
Agreeableness	48 (5)	44 (6)	*t* = −2.42*
Conscientiousness	46 (6)	43 (6)	*t* = −2.08*

*Note*: Numbers present means (standard deviations) unless indicated otherwise.

^a^ *n* = 17, as six non‐musicians never started musical training.

* *p* < 0.05.

*** *p* < 0.0001.

Cognitive and affective empathy were moderately positively correlated (*r* = 0.37, *p* < 0.01). Cognitive empathy was moderately positively correlated with openness (*r* = 0.39, *p* < 0.006), conscientiousness (*r* = 0.36, *p* < 0.02) and agreeableness (*r* = 0.33, *p* < 0.03). Affective empathy was strongly positively correlated with agreeableness (*r* = 0.60, *p* < 0.001).

### 3.2 Preliminary Analyses on the EAT Data

EA did not significantly differ with the gender of the target, *F*(1, 47) = 0.18, *p* > 0.6, *d =* 0.13. However, EA differed with the valence of the video clips, *F*(1, 47) = 62.99, *p* < 0.0001, *d* = 2.34. As expected, based on several previous studies, participants obtained higher EA with positive clips (mean *r* = 0.73) than with negative clips (mean *r* = 0.57). Thus, we considered Valence as a covariate during hypothesis testing.

### 3.3 Hypothesis Testing: Group Differences in EA

The main effect for Group was not significant when it was the sole predictor, *F*(1, 46) = 1.59, *p* > 0.2, *d* = 0.37. When we added Valence as a covariate, this result did not change. When we added Valence as a moderator instead, there was no significant Group by Valence interaction, *F*(1, 46) = 0.22, *p* > 0.61. Post hoc, we decided to also examine the main effect for Group for positive and negative video clips separately. In this unplanned analysis, it was also not significant (positive clips: *F*(1, 46) = 0.39, *p* > 0.5, *d* = 0.18, negative clips: *F*(1, 46) = 1.70, *p* > 0.1, *d* = 0.38). In sum, there was no evidence for a significant group difference in EA.

### 3.4 Additional Analyses

As musicians reported higher levels of cognitive and affective empathy on the EQ, as well as higher levels of three Big Five personality traits, we examined the predictive value of these variables for EA and their impact on potential group differences on the EAT. Models and outcomes are summarised in Tables S1 and S2. Cognitive empathy and affective empathy did not significantly predict EA, nor did Big Five openness, agreeableness, and conscientiousness. Moreover, the main effect for Group and the interaction effects involving Group were never significant.

Relevant musical background questionnaire variables were also examined as predictors of EAT performance. Models and outcomes are summarised in Table S3. Having started musical training at a younger age predicted higher EA, *p* < 0.04. Length of musical training and the weekly amount of music making were not significant predictors. Moreover, the main effect for Group and the interaction effects involving Group were never significant.

## 4 Discussion

The main finding of our study was that there was no convincing evidence of a difference in empathy between musicians and non‐musicians when using the EAT, a performance measure, even though musicians rated themselves higher on the EQ, a self‐report questionnaire. Relatedly, participants' EA scores were not significantly predicted by their EQ subscale scores. Moreover, EA scores were not significantly predicted by their scores on relevant NEO‐FFI subscales, and could thus not be linked to the Big Five personality traits.

The EAT requires participants to rate the emotions of others narrating autobiographical positive and negative events. Our result of no significant group difference on this task is in line with findings of Morand‐Grondin et al. (2025), published after our study was completed. Their sample of 80 adults with and 89 adults without musical experience completed two other performance measures of empathy, the Reading the Mind in the Eyes Test (RMET) and the Multifaceted Empathy Test (MET). Morand‐Grondin et al. (2025) found no significant group differences on either measure. Of note, both measures use photo stimuli instead of video clips, which means there is no audio component, unlike in the EAT used in our study.

Morand‐Grondin et al. (2025) also asked participants to complete the Basic Empathy Scale, a self‐report questionnaire. While they also found no significant group difference on this measure, musicians in our study had a higher score on the self‐report questionnaire we included, the EQ. This inconsistency is in line with our finding that the EQ did not significantly predict EAT performance, which in turn is in line with a meta‐analysis of 85 studies showing that self‐report measures of cognitive empathy do not reliably predict empathic abilities assessed using performance‐based measures (Murphy and Lilienfeld 2019).

Beyond this, our finding that musicians did not objectively perform better on the EAT, even though they subjectively rated themselves as more empathic on the EQ, has several possible explanations. First, the musicians in our sample (who were mostly pianists, string players, or singers) may have been overrating their level of empathy on the EQ. Butkovic and Modrusan (2021) found that their musician participants, in particular the pianists, string players and singers who were studied, rated their own agreeableness and openness higher than their peers did. These findings support the possibility that musicians in our study were relatively likely to rate themselves as more empathetic than they actually were.

Second, the null results for the EAT but not the EQ may be taken to mean that EA is less sensitive to between‐group variation than subjectively assessed empathy. This idea might be supported by other studies reporting null results on a performance measure but not on a self‐report measure of empathy. Indeed, McKenzie et al. (2022) found differences between individuals with vs. without autism spectrum disorder on self‐reported empathy but not on the EAT. Nonetheless, other studies have found group differences in EA, for example when studying individuals with vs. without schizophrenia (Lee et al. 2011).

Third, the absence of a significant group difference in EA in our study may be explained by the fact that the EAT provides participants with relatively long video clips that are rich in both auditory and visual information, containing prosodic cues, semantic content, and facial and postural expressions. Hence, any benefit musicians may have in the domain of VER and emotional prosody perception (Jansen et al. 2023; Martins et al. 2021) might have been diluted due to the overall richness of the EAT stimuli. Relatedly, Martins et al. (2021) found that differences between musicians and non‐musicians are more often observed in VER studies than in facial emotion recognition studies. Musicians may be better at VER because they have learnt to process prosodic acoustic cues better (Fuller et al. 2014; Jansen et al. 2023) but this ability may not generalise to the visual domain. This implies that musicians might have shown higher EA if we had used an audio‐only version of the EAT, particularly while listening to more expressive targets, who provide more prosodic information (Zaki et al. 2009).

Finally, it is possible that a potential musician benefit in empathy may be captured more easily by a measure that relies on emotional expressions in music than by a measure that involves speech narratives covering autobiographical emotional events. For example, Wöllner (2012) described a method that involved trained musicians watching film clips of individuals playing in a quartet and rating each individual's emotional expressiveness; comparable to the EAT, each person in the quartet had previously also rated their own expressiveness. In another study, participants completed an EAT as well as a comparable (but more difficult) task involving emotional music stimuli rather than video clips (Tabak et al. 2023). Accuracy on the music task was positively predicted by participants' self‐reported affective empathy in the first study but not in the second study. However, neither study compared musicians to non‐musicians. Nonetheless, music task accuracy was not significantly associated with participants' duration of musical training in the first study nor with self‐reported musicality in the second study. This contradicts the idea that a musician benefit might be more easily captured by an emotional music task.

Overall, we believe that firm conclusions about whether musicians and non‐musicians differ in empathy can only be drawn after follow‐up research. We offer three recommendations. First, considering the questionable internal consistency of the Affective Empathy subscale of the EQ in our sample, we suggest including the Questionnaire of Cognitive and Affective Empathy (QCAE; Reniers et al. 2011; Wöllner 2012) instead. Unlike the EQ (and the IRI; Novembre et al. 2019), for which the subscales were derived post hoc, the QCAE was designed a priori to tap into both empathy components. Second, we suggest comparing the regular EAT with an audio‐only version, as this could support previous findings of higher VER in musicians, yet also show that visual information is usually so rich that in everyday situations empathy does not require good VER. More specifically, while Zaki et al. (2009) previously studied an audio‐only version that included both prosodic and semantic information, we believe a prosody‐only version might most likely show the expected group difference. Third, we suggest also administering visual‐only emotion recognition measures as control measures (Suslow et al. 2023).

### 4.1 Study Limitations

Not only the EQ Affective Empathy subscale had questionable internal consistency, but also the NEO‐FFI Agreeableness subscale. Moreover, the internal consistency of the NEO‐FFI Openness subscale was poor. These subscales may have been measuring something else instead of, or in addition to, the constructs we intended them to measure. This limits our ability to link our NEO‐FFI results to previous studies showing higher agreeableness and openness in musicians (Butkovic and Rancic Dopudj 2017; Gjermunds et al. 2020).

Another limitation is that we did not ask musicians how often they were taking part in collective music‐making. Enhanced empathy in musicians may be more pronounced in musicians making music collectively than in musicians making music alone. This idea stems from correlational research showing that music students who participate in collective music‐making more often show a higher level of empathy (Cho and Yeoun Han 2022).

### 4.2 Implications for Future Research

Post hoc, among participants with at least some musical training (i.e., the musicians and most of the non‐musicians), we found higher EA if they had started musical training at a younger age. This could mean that starting musical training young may contribute to enhanced EA, which is relevant from a developmental perspective. Musical training in childhood might have a larger impact on empathy, or on underlying processes such as VER and emotional prosody perception, than having musical training equally long in adulthood. Notably, Morand‐Grondin et al. (2025) also reported higher RMET and MET performance among participants who started musical training at a younger age. Both are objective measures, like the EAT and unlike the EQ (which in previous research did not correlate with the age musical training started; Cho and Yeoun Han 2022). Unlike the RMET and MET, however, the EAT relies on both visual and auditory stimuli, so it remains possible that musical training might have an impact on empathy via both visual and auditory processes.

A future study in musicians could examine this by using multiple measures of empathy, VER, and emotional prosody perception. We recommend matching musicians (a) to a group of non‐musicians based on the age they started musical training, regardless of the duration of subsequent training, and (b) to a group of non‐musicians based on the duration of training, regardless of when they started it. To minimise the risk that the type of training is a confounding variable, we also recommend that all participants play the same or a comparable instrument, and are primarily engaged in either individual or group musical activities.

## 5 Conclusions

Musicians were found to differ from non‐musicians on a self‐report measure of empathy, the EQ, but not on a performance‐based measure, the EAT. While musicians may have enhanced VER and emotional prosody perception (Jansen et al. 2023; Martins et al. 2021), they did not benefit from this during the EAT. As the stimuli used in the EAT contain not only auditory information but also dynamic visual cues, non‐musicians may have compensated for their potentially lower auditory abilities by focusing on the other information provided.

The EAT is considered a more objective and ecologically valid empathy measure than self‐report empathy measures (aan het Rot and Hogenelst 2014; Zaki et al. 2008). Consequently, musicians' higher subjective empathy is more likely related to third variables that potentially led them to start musical training than to a causal effect of musical training. As musicians' higher self‐reported empathy did not extend to actual performance in the lab, it is questionable that musicians would exhibit greater empathy than non‐musicians in the real world.

## Ethics Statement

The present study was positively reviewed by the Ethics Committee of the Department of Psychology at the University of Groningen (file number 1819‐S‐0007). The study was conducted in accordance with ethical standards comparable to the Declaration of Helsinki.

## Consent

All participants provided written informed consent and were remunerated for their time according to departmental guidelines.

## Conflicts of Interest

The authors declare no conflicts of interest.

## Supporting information


**Data S1.** Supporting Information.

## Data Availability

The anonymized data that support the findings of this study are available via DataverseNL at https://doi.org/10.34894/HVC0P7.
