# Supplementary material for: Empathy in Musicians: Self‐Report Versus Performance on an Empathic Accuracy Task
Source: Int J Psychol. 2025 Jul 11;60(4):e70072. doi: 10.1002/ijop.70072 (PMC12254598; doi:10.1002/ijop.70072)
Supplement: Supplementary file 1 — Data S1. Supporting Information. [file IJOP-60-e70072-s001.docx]

Supplementary Table 1. Outcomes of models examining cognitive or affective empathy as moderators for group differences in empathic accuracy

| Predictors | Cognitive empathy | Affective empathy | Group | Cognitive empathy * Group | Affective empathy * Group |
| --- | --- | --- | --- | --- | --- |
| Model 1a | 2.31 |  |  |  |  |
| Model 1b |  | 0.78 |  |  |  |
| Model 2 | 0.64 | 0.13 |  |  |  |
| Model 3a | 0.98 |  | 0.28 |  |  |
| Model 3b |  | 0.80 | 1.09 |  |  |
| Model 4a | 0.79 |  | 2.27 | 2.95 |  |
| Model 4b |  | 0.38 | 0.07 |  | 0.24 |

Note. Values represent *F* statistics.

Supplementary Table 2. Outcomes of models examining Big Five openness, agreeableness, and conscientiousness as moderators for group differences in empathic accuracy

| Predictors | Openness | Agreeableness | Conscientiousness | Group | Openness * Group | Agreeableness * Group | Conscientiousness * Group |
| --- | --- | --- | --- | --- | --- | --- | --- |
| Model 1a | 0.00 |  |  |  |  |  |  |
| Model 1b |  | 0.51 |  |  |  |  |  |
| Model 1c |  |  | 0.00 |  |  |  |  |
| Model 2 | 0.00 | 0.56 | 0.04 |  |  |  |  |
| Model 3a | 0.22 |  |  | 1.82 |  |  |  |
| Model 3b |  | 0.10 |  | 1.17 |  |  |  |
| Model 3c |  |  | 0.14 | 1.74 |  |  |  |
| Model 4a | 0.25 |  |  | 0.20 | 0.09 |  |  |
| Model 4b |  | 0.09 |  | 0.35 |  | 0.21 |  |
| Model 4c |  |  | 0.09 | 0.39 |  |  | 0.20 |

Note. Values represent *F* statistics.

Supplementary Table 3. Outcomes of models examining relevant musical background variables as moderators for group differences in empathic accuracy

| Predictors | Age at start musical training | Length of musical training | Weekly amount of music making | Group | Age at start musical training * Group | Length of musical training * Group | Weekly amount of music making * Group |
| --- | --- | --- | --- | --- | --- | --- | --- |
| Model 1a | 4.58* |  |  |  |  |  |  |
| Model 1b |  | 1.08 |  |  |  |  |  |
| Model 1c |  |  | 0.30 |  |  |  |  |
| Model 2 |  |  |  |  |  |  |  |
| Model 3a | 3.34† |  |  | 0.02 |  |  |  |
| Model 3b |  | 0.18 |  | 0.69 |  |  |  |
| Model 3c |  |  | 0.26 | 1.55 |  |  |  |
| Model 4a | 0.02 |  |  | 0.64 | 0.85 |  |  |
| Model 4b |  | 0.05 |  | 2.49 |  | 1.83 |  |
| Model 4c |  |  | 0.92 | 0.74 |  |  | 0.70 |

Note. Values represent *F* statistics. *p<0.05, †p<0.1.
